# Heartland Virus Neutralizing Antibodies in Vertebrate Wildlife, United States, 2009–2014

**DOI:** 10.3201/eid2110.150380

**Published:** 2015-10

**Authors:** Kasen K. Riemersma, Nicholas Komar

**Affiliations:** Centers for Disease Control and Prevention, Fort Collins, Colorado, USA

**Keywords:** Heartland virus, Heartland disease, Phlebovirus, Bunyaviridae, Amblyomma americanum, vertebrate wildlife serosurvey, zoonoses, ticks, viruses, vector-borne infections, United States

## Abstract

Since its discovery in 2009, the tickborne Heartland virus (HRTV) has caused human illness in Missouri, Oklahoma, and Tennessee USA. To better assess the geographic distribution of HRTV, we used wildlife serology as an indicator. This retrospective evaluation determined that HRTV is widespread within the central and eastern United States.

Heartland virus (HRTV; family *Bunyaviridae*, genus *Phlebovirus*) is an emerging public health threat in the United States. HRTV disease, characterized by severe fever, leukopenia, and thrombocytopenia, was first reported in 2 farmers in northwestern Missouri in 2009 (*1*). Seven additional HRTV disease cases (2 fatal) have been reported in Missouri, Tennessee, and Oklahoma (*2*,*3*). A study of ticks and mosquitoes in northwestern Missouri detected HRTV infections only in *Amblyomma americanum* (lone star tick) and thus implicated this tick as a vector (*4*). The virus was isolated solely from deplete host-seeking nymphs, which presumably were infected as larvae after feeding on a viremic vertebrate host. Because HRTV has yet to be isolated from any wild or domestic animals, the question of vertebrate reservoir(s) remains unanswered. However, high prevalence of seropositive white-tailed deer (*Odocoileus virginianus*) and raccoon (*Procyon lotor*) from northwestern Missouri indicate these species as targets for wildlife serosurveillance (*5*).

The HRTV disease case reports in Tennessee and Oklahoma after the initial case reports in Missouri create the perception that HRTV transmission activity might be dispersing from an origin in northwestern Missouri. However, the geographic range of HRTV activity is unknown. HRTV distribution may mirror the range of the lone star tick, which is distributed throughout most of the central and eastern United States and recently has expanded northward (*6*). To investigate the hypothesis that HRTV activity occurs throughout the range of its putative tick vector, we conducted a retrospective serosurvey of mainly white-tailed deer and raccoon from 19 states within the heart and periphery of the lone star tick range to look for evidence of HRTV activity. 

## The Study

Banked blood samples collected from white-tailed deer, raccoon, and (occasionally) moose (*Alces alces*) and coyote (*Canis latrans*) during 2009–2014 were analyzed by plaque-reduction neutralization test for HRTV neutralizing antibodies by using African green monkey kidney (Vero) cell culture. Only samples from healthy live-trapped animals or deceased animals from anthropogenic causes (i.e., hunting, culls, and automobile strikes) were tested. Inclusion of 19 states was opportunistic based on sample availability. We used the plaque-reduction neutralization test to evaluate HRTV seropositivity for white-tailed deer (n = 396), raccoon (n = 949), coyote (n = 61), and moose (n = 22) (Table 1). Samples consisted of whole blood dried onto Nobuto strips (Advantec MFS, Inc., Dublin, CA, USA), bloody body cavity fluids, or hemolyzed whole blood. Nobuto strip samples were eluted to 1:10 serum dilution in phosphate buffer solution in accordance with the manufacturer’s instructions. All samples were heat-inactivated at 56°C for 45 min.

**Table 1:** Animals screened and confirmed seropositive for Heartland virus neutralizing antibodies, central and eastern United States, 2009–2014

State	No. (%) counties sampled	Species	No. screened	Confirmed seropositive, no. (%; 95% CI)
Alabama	5 (7)	Raccoon (*Procyon lotor*)	99	0 (0; 0–4)
Florida	34 (51)	White-tailed deer (*Odocoileus virginianus*)	65	4 (6; 2–15)
		Raccoon	40	0 (0; 0–9)
Georgia	1 (1)	White-tailed deer	104	15 (14; 8–23)
Illinois	8 (8)	Coyote (*Canis latrans*)	25	1 (4; 1–20)
		Raccoon	68	0 (0; 0–5)
Indiana	13 (14)	Raccoon	64	2(3; 1–11)
Iowa	6 (6)	Coyote	2	0 (0; 0–5)
		Raccoon	98	0 (0; 0–13)
Kansas	10 (10)	Coyote	22	10 (46; 27–65)
Kentucky	7 (6)	Raccoon	44	4 (9; 4–21)
Maine	6 (38)	White-tailed deer	63	7 (11; 6–21)
Missouri	10 (9)	Coyote	12	0 (0; 0–24)
		White-tailed deer	2	0 (0; 0–66)
		Raccoon	75	10 (13; 7–23)
New Hampshire	7 (70)	Moose (*Alces alces*)	22	4 (18; 5–40)
		White-tailed deer	58	9 (16; 7–27)
North Carolina	2 (2)	White-tailed deer	32	13 (41; 24–59)
Ohio	7 (8)	Raccoon	94	0 (0; 0–4)
Pennsylvania	15 (22)	Raccoon	81	0 (0; 0–5)
Tennessee	7 (7)	Raccoon	92	13 (14; 8–23)
Texas	22 (9)	Raccoon	85	4 (5; 2–12)
Vermont	5 (36)	White-tailed deer	72	7 (10; 5–19)
Virginia	2 (2)	Raccoon	37	0 (0; 0–9)
West Virginia	19 (35)	Raccoon	72	0 (0; 0–5)
Total			1,428	103 (7; 6–9)

We screened the inactivated samples at 1:20 dilution by mixing serum diluted 1:10 with equal volume of titrated HRTV to approximate a challenge dose of 50 PFUs. Treated Vero cells were incubated for 1 h at 37°C, 5% CO_2_, before applying a nutrient-rich 0.5% agarose overlay. A second overlay containing Neutral Red was applied after 5–7 d of incubation. Viral plaques were counted 6–12 d after inoculation. A neutralization threshold of 70% relative to HRTV-only controls was used to determine positive samples. All screen-positive samples were repeat-tested to confirm results. Samples were considered seropositive if they were confirmed as positive at a dilution of >1:40. Comparative neutralization tests with related viruses were not performed, because we had previously found that murine antiserum developed against the other known phleboviruses in the United States—Sunday Canyon virus (*7*), Rio Grande virus (*8*), and Lone Star virus (*9*)—had no appreciable neutralizing activity against HRTV (Table 2). Human antiserum developed against HRTV exhibited weak 1-way neutralization of Lone Star virus and Sunday Canyon virus (Table 2).

**Table 2:** Lack of detectable cross-neutralization of HRTV by mouse hyperimmune ascites fluids containing high-titered antibodies to LSV, SCV, and RGV*

Virus (challenge dose, PFU)	PRNT_70_ antibody titers			
	HRTV	LSV	SCV	RGV
HRTV (54)	160	<20	<20	<20
LSV (214)	20	≥640	<20	<20
SCV (220)	20	<20	320	<20
RGV (14)	<20	<20	<20	320

*HRTV, Heartland virus; LSV, lone star virus; PRNT_70_, 70% plaque-reduction neutralization test; RGV, Rio Grande virus; SCV, Sunday Canyon virus.

**Figure 1:**
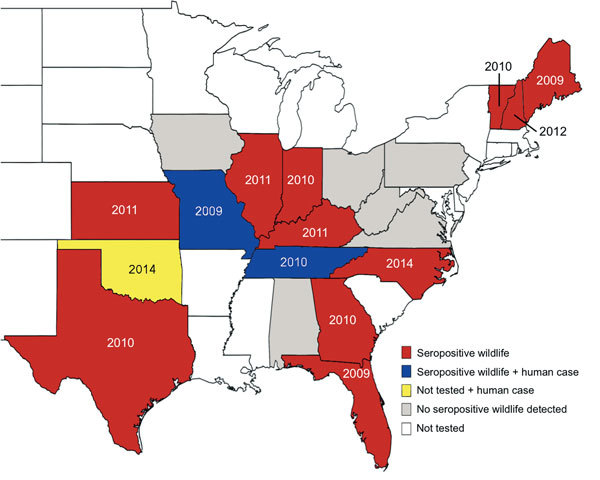
State-level distribution of Heartland virus case reports in humans and seropositive wildlife, central and eastern United States, 2009–2014. Red indicates states with seropositive animals; gray indicates states with no seropositive animals. Year labels indicate the earliest year of detected HRTV activity. Earliest detection was determined by human case reports in Missouri (1 case) and Oklahoma (3 cases) and wildlife serologic data in all other states.

**Figure 2:**
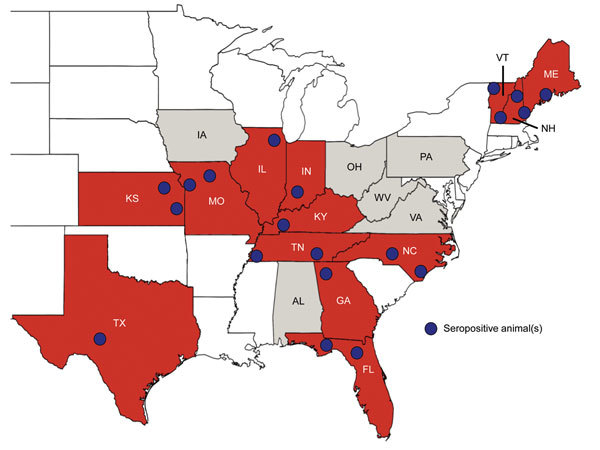
Geographic groupings of confirmed seropositive animals for Heartland virus neutralizing antibodies, central and eastern United States, 2009–2014. Twenty groups were identified in 13 states. The geographic locations of the groups were subjectively approximated by the counties where seropositive animals were collected (blue circles). Red indicates states with seropositive animals; gray indicates states in which no seropositive animals were detected. Because of the sampling design, the data are qualitative.

Of 1,428 animals, 103 were seropositive: 55 deer, 33 raccoon, 11 coyotes, and 4 moose. Thirteen states had seropositive animals: Florida, Georgia, Illinois, Indiana, Kansas, Kentucky, Maine, Missouri, New Hampshire, North Carolina, Tennessee, Texas, and Vermont (Table 1; Figure 1). Within the 13 states, 20 geographic clusters of seropositive animals were mapped by plotting positive animals by the county where they were collected (Figure 2).

## Conclusions

We provide evidence of widespread HRTV transmission activity across the central and eastern United States. Of 13 affected states, only Missouri and Tennessee had previous evidence of HRTV activity. A more stringent neutralization threshold of 80% would reclassify 14 positive samples to “equivocal,” but the number of positive states would remain unchanged. These findings should encourage clinicians and public health officials to consider HRTV as a potential source of illness throughout the eastern United States.

Surprisingly, seropositive white-tailed deer were detected in northern New England, where established populations of lone star ticks are unknown (*6*). Possible explanations include unreported lone star tick populations, immigration of seropositive deer, alternative tick vectors for HRTV, or presence of a serologically cross-reactive virus. Movement of deer across state boundaries is an unlikely explanation. Extensive lone star tick populations are not reported in neighboring states (*6*), and migration of deer from lone star tick–infested regions is unlikely (*10*). Savage et al. did not detect HRTV RNA in *Dermacentor variabilis*, the American dog tick (*4*), but additional tick species inhabit northern New England. Several tick species are reported to transmit severe fever with thrombocytopenia syndrome virus, a closely related phlebovirus found in eastern Asia (*11*). Further investigation of tick populations and their vector competence for HRTV is warranted, and production of HRTV neutralizing antibodies in response to a serologically similar virus should be investigated. Two new phleboviruses recently detected in *Ixodes* ticks in the northeastern United States are genetically unrelated to HRTV but raise the possibility that additional undiscovered phleboviruses exist (*12*). Severe fever with thrombocytopenia syndrome virus–reactive antibodies in wildlife were reported in Minnesota, also peripheral to the lone star tick geographic range, indicating the likely presence of HRTV or a similar virus there (*13*).

The finding of seropositive moose and coyotes indicates that these mammals are exposed to HRTV in certain situations and might be useful targets for serosurveillance, in addition to deer and raccoon. The full vertebrate host range and the reservoir competence of these mammals for HRTV remains unknown.

The chronology of dispersal of HRTV is unclear. Suggesting that HRTV emerged in northwestern Missouri and spread to neighboring states to the east and south is overly simplistic. Because animals were sampled at different points of time and space during this study, our data lack robustness to enable comparison of populations over time or between geographic locations. Thus, we are unable to evaluate the dynamics of HRTV spread. Furthermore, the proportions of HRTV-seropositive animal populations lack quantitative value because of our retrospective convenience sampling. Our results simply indicate that HRTV or a very similar virus has circulated in the sampled regions in the recent past and that this activity began as early as 2009. Adult seropositive white-tailed deer were detected in Maine and Florida in 2009, and based on the estimated ages of affected deer (data not shown), the infections could have occurred as early as 2003. A much larger retrospective serosurvey is necessary to elucidate HRTV’s history of emergence.
